# Intact and cleaved forms of the urokinase receptor enhance discrimination of cancer from non-malignant conditions in patients presenting with symptoms related to colorectal cancer

**DOI:** 10.1038/sj.bjc.6605228

**Published:** 2009-08-11

**Authors:** A F Lomholt, G Høyer-Hansen, H J Nielsen, I J Christensen

**Affiliations:** 1 Department of Surgical Gastroenterology, Copenhagen University Hospital Hvidovre, 30 Kettegaard Allé, DK-2650 Hvidovre, Denmark; 2 The Finsen Laboratory, Copenhagen Biocenter, 5 Ole Maaloes Vej, DK-2200 Copenhagen N, Denmark

**Keywords:** uPAR, colorectal cancer, detection

## Abstract

**Background:**

Colorectal cancer (CRC) is a leading cause of cancer-related morbidity and mortality in developed countries. It is known that early detection results in improved survival, and consequently there is a need for improved diagnostic tools in CRC. The plasma level of soluble urokinase plasminogen activator receptor (suPAR) was proposed as a marker in CRC patients. This study was undertaken to evaluate the individual molecular forms of suPAR as discriminators in a group of patients undergoing endoscopical examination following symptoms related to colorectal cancer.

**Methods:**

In a case–control study comprising 308 patients undergoing endoscopical examination following CRC-related symptoms, 77 CRC patients with adenocarcinoma were age and gender matched to: 77 patients with adenomas; 77 with other non-malignant findings, and 77 with no findings. The different uPAR forms were measured in citrate plasma collected before endoscopical examination, using three different Time Resolved – Fluorescence Immuno Assays (TR-FIA's).

**Results:**

All soluble uPAR forms were found to be significantly higher in cancer patients than in patients presenting with other non-malignant findings; uPAR(I) *P*=0.0006, suPAR(I–III) *P*<0.0001 and suPAR(I–III)+(II–III) *P*<0.0001, whereas no significant difference was found when performing similar comparisons for patients presenting with adenomas. The odds ratio (OR) for the comparison of uPAR(I) in patients with CRC to subjects with other non-malignant findings was 3.44 (95% CI:1.86–6.37). CRC patients had a mean elevated level of 20.9% (95% CI:10.2–32.6) for suPAR(I–III) and 18.5% (95% CI:9.0–28.8) for suPAR(I–III)+(II–III) compared with subjects with non-malignant findings.

**Conclusions:**

The findings confirm reports on increased uPAR expression in cancer patients and in particular elevated levels of suPAR in blood from CRC patients and indicate that suPAR levels in blood are increasing during carcinogenesis. Although none of the measured uPAR forms were cancer specific, our findings suggest that uPAR expression could be useful in the early detection of CRC when combined with other markers and clinical variables.

Colorectal Cancer (CRC) is a leading cause of cancer with an estimated individual lifetime risk of 5% in developed countries. Worldwide CRC accounts for approximately 1 million new cases per year, and in Europe CRC is the second most prevalent cancer and the second most important in relation to cancer-specific death (Parkin *et al*, 2005; Ferlay *et al*, 2007). Outcome in CRC patients relates to the stage of disease at diagnosis, and because more than 50% of patients are diagnosed at a late stage of disease, there is a need for new diagnostic tools for early detection of CRC (Weitz *et al*, 2005). According to The World Health Organization conditions for early detection should be an appropriate disease-control approach: (1) concerning a common disease with serious morbidity and mortality; (2) potential screening tests should be able to accurately detect early-stage disease; (3) treatment after detection should be proven to improve prognosis relative to usual diagnosis; and (4) evidence should exist that the potential benefits outweigh the potential harms and costs of screening (Winawer *et al*, 1990). In this respect CRC has been proposed as an ideal screening object, but so far no ideal screening method has been found. Presently the ‘golden standard’ for screening of patients at risk of CRC is colonoscopy, as the incidence of CRC in screening populations undergoing colonoscopy has been shown to decrease (Winawer *et al*, 1993; Levin *et al*, 2008). However, this procedure is time consuming, expensive, inconvenient for the patient and includes a small risk of perforation, and consequently colonoscopy is not ideal for primary population-based screening. The Faecal Occult Blood Test (FOBT) was proposed as a potential screening tool even though the sensitivity and specificity of this test were shown to vary considerably in different studies, additionally low compliance impose problems for the use of FOBT in CRC population-based screening (Hewitson *et al*, 2006; Burch *et al*, 2007). The search for molecular markers in blood has been promising and multiple markers have been proposed, but limited reproducibility results in bias emphasizing the need for studies designed for this purpose (Ransohoff, 2007). No serological marker has yet been accepted for use in early detection of CRC (Duffy *et al*, 2007), even though some serological biomarkers, among these the urokinase receptor, uPAR, have proven to be strong prognostic markers in CRC (Stephens *et al*, 1999).

The plasminogen activation system (PA-system) is involved in tissue remodelling processes, including tumour invasion and metastasis (Werb, 1997; Irigoyen *et al*, 1999; Andreasen *et al*, 2000). Components of this system including the urokinase plasminogen activator (uPA), its receptor uPAR and the uPA inhibitor (PAI-1), have been shown to be elevated in tumour tissue and blood from cancer patients including patients with CRC (Ganesh *et al*, 1994, 1996; Pedersen *et al*, 1994; Stephens *et al*, 1999; Mustjoki *et al*, 2000; Riisbro *et al*, 2002; Hoyer-Hansen and Lund, 2007).

uPAR has a central function, because binding of the zymogen pro-uPA initiates activation of cell surface bound plasminogen leading to other proteolytic events in the extracellular matrix (Ellis *et al*, 1989). UPAR(I–III) consists of three domains denoted uPAR(I), uPAR(II) and uPAR(III) connected by two linker regions, and the crystal structure has been solved (Llinas *et al*, 2005). uPAR(III) is attached to cell membrane by a glycosyl–phosphatidyl–inositol (GPI) anchor(Ploug *et al*, 1991). Intact uPAR is required for efficient binding of uPA (Hoyer-Hansen *et al*, 1997). uPAR(I–III) can also be cleaved in the linker region between domains I and II by uPA, liberating uPAR(I) and leaving the cleaved form, uPAR(II–III) on the cell surface (Hoyer-Hansen *et al*, 1992). uPA has been shown to cleave uPAR *in vivo* (Zhou *et al*, 2000). uPAR is shed from the cell surface and soluble forms of uPAR: suPAR(I–III), suPAR(II–III) and uPAR(I) have been detected in various body fluids (Hoyer-Hansen and Lund, 2007). Although the mechanism of uPAR shedding is not clarified, evidence has been provided that the glycolipid (GPI) anchor can be cleaved by endogenous cellular GPI-specific phospholipase D (GPI–PLD) (Wilhelm *et al*, 1999). Although GPI-anchored uPAR(I–III) is readily cleaved by uPA, suPAR(I–III) cannot be cleaved by uPA in the linker region between domains I and II (Hoyer-Hansen *et al*, 2001; Hoyer-Hansen and Lund, 2007). Thus, uPAR(I) detected in blood results from cell surface cleavage of uPAR(I–III) and suPAR(II–III) is in addition shed from the cell surface.

In colon cancer uPAR is expressed by tumour-infiltrating macrophages and fibroblasts and by a few cancer cells (Pyke *et al*, 1994; Ohtani *et al*, 1995). Total levels of all uPAR forms in tumour extracts and as before mentioned in blood have been shown to correlate with poor prognosis in patients with colorectal cancer (Ganesh *et al*, 1994; Stephens *et al*, 1999; Konno *et al*, 2001). We hypothesized that cleavage of uPAR is an indication of an active plasminogen activation system and that the amounts of the individual cleavage products would consequently be superior to the total uPAR level as diagnostic markers of cancer. To investigate this we employed immunoassays selectively quantifying the individual uPAR forms (Piironen *et al*, 2004). Using these assays we have previously shown that the serum level of uPAR(I) enhances the discrimination of benign from malignant prostatic disease (Piironen *et al*, 2006). In addition, uPAR(I) was found to be a stronger prognostic marker than the total amount of uPAR in non-small cell lung cancer (Almasi *et al*, 2005). In a recent study, we also showed that suPAR(I) in blood enhances discrimination of benign from malignant disease and uPAR(I) in blood predicts survival in patients with ovarian cancer (Henic *et al*, 2008).

This case–control study was undertaken to evaluate the potential discriminatory value of the plasma uPAR variants in four diagnostic groups of patients undergoing endoscopical examination following symptoms related to CRC.

## Subjects and methods

### Subjects

From October 2003 through December 2005, subjects were included in a multi-centre cross sectional study conducted at six Danish hospitals. Eligible for inclusion were patients (aged 18+ years) undergoing endoscopical examination following symptoms related to CRC and patients attending surveillance programs due to Hereditary Colorectal Cancer (HNPCC and FAP). Subjects previously diagnosed with CRC and subjects unable to give informed consent were excluded from the study. The Regional Ethical Committee approved the study. Following oral and written consent according to the Helsinki II Declaration, 5165 individuals were included consecutively – for details see (Nielsen *et al*, 2007). Based on this study population a case–control study was designed including 312 patients representing four diagnostic groups of subjects. This study population was selected to test the diagnostic potential of different promising serological markers in CRC. Primarily, 78 subjects with pathologically verified colorectal adenocarcinomas (25% RC, *n*=20 and 75% CC, *n*=58) were selected at random. For each of these, a subject with pathologically verified adenomatous changes was randomly selected matching for age, gender and localisation of finding. Then subjects with other (non-malignant) findings were randomly selected and matched as described for the adenomas, and finally subjects were randomly selected who had no findings at endoscopy and who reported no co-morbidity. All pathological diagnoses were validated using the Danish National Registry on Pathological Examinations (www.patobank.dk). Patients who were diagnosed with cancer but did not have the tumour removed were classified according to the clinical information available, and as a result exact tumour staging was not possible in all cases. Similarly for patients diagnosed with adenomas, the type of adenoma was not specified in all cases, either because of incompleteness of the pathological description available or due to loss for specimen during the examination. Previous cancer diagnoses were retrieved from the Danish Cancer Registry, one subject had a previous CRC and was excluded from the study together with three matched subjects, leaving a study population of 308 subjects. Descriptive statistics for the matched subjects are given in Table 1.

### Sampling

Blood samples were collected before endoscopical examination in all subjects, following a standard operating procedure (Nielsen *et al*, 2007).

At sample collection subjects were non-fasting. Patients undergoing colonoscopy had completed a bowel preparation. Blood was collected at moderate tourniquet pressure; in 4 ml citrate-coated tubes (Vacutainer Becton-Dickinson, Mountain View, CA, USA), and spun for 10 min at 2500 *g* and 4 °C within 1 h following collection. Plasma was collected and samples were immediately stored at −80 °C and were thawed and refrozen once before analysis.

### Immunoassay

Samples were analysed using three different Time Resolved Fluorescence Immuno Assays (TR-FIAs). TR-FIA 1 measuring full-length suPAR(I–III), TR-FIA 2 measuring full-length and cleaved suPAR(I–III)+(II–III), and TR-FIA 3 measuring liberated domain I, uPAR(I). All samples were analysed in a 1:5 dilution. The assays and their validation in citrate plasma have previously been described (Henic *et al*, 2008). In all three settings samples were read in the Floustar Galaxy fluorometer with excitation set at 405 nm and emission read at 615 nm with a 400 *μ*s delay and a 400 *μ*s acquisition window.

### Statistics

Descriptive statistics are presented by the median, minimum, maximum or frequency if applicable. The Spearman's rank correlation was used as a measure of association. The statistical analyses were based on a case–control design. For suPAR(I–III) and suPAR(I–III)+ (II–III) a normal distribution was assumed following log-transformation (natural). Domain I (uPAR(I)) was treated as a binary variable using the limit of quantification of the assay as the cut-point. Primarily a test for association between the serological marker and CRC, adenoma, ONM findings and no findings (hereafter denoted diagnosis groups) was carried out. The analysis was done using a general linear model with diagnosis group as the explanatory variable and the serological marker as the dependent variable. For markers normally distributed on the log scale, the link function used was the identity function and for dichotomized serological markers, the logit was chosen as the link function. The analysis used repeated measures with subject being each case with corresponding samples. For each case, the proportion of cancer patients with uPAR levels exceeding the level found in the corresponding adenoma, ONM and no finding was estimated with 95% CI.

Database management and statistical calculations were done using SAS (v 9.1, SAS Institute, Cary, NC, USA). *P*-values below 5% were considered significant.

## Results

**Figure 1:**
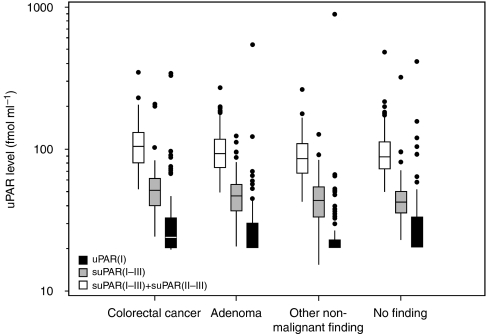
Box plot showing the distribution of the different plasma uPAR forms in the four different diagnostic groups.

Patient characteristics for the 77 matched subjects including age; gender and localization of findings are given in Table 1. In Figure 1 plasma levels of the different uPAR forms are depicted for each diagnostic group. All soluble uPAR forms were found to be significantly higher in cancer patients than in patients presenting with ONM findings; uPAR(I) *P*=0.0006, suPAR(I–III) *P*<0.0001 and suPAR(I–III)+(II–III) *P*<0.0001. Comparisons between the group of patients presenting with adenomas and the remaining diagnostic groups showed no significant difference.

### Association between the different suPAR variants

To evaluate the association between the different suPAR variants the Spearman's rank coefficient was calculated. In all cases a significant correlation between the different suPAR variants was shown p<0.0001 (data not shown). The weakest correlation was found comparing uPAR(I) and suPAR(I–III) resulting in a 0.60 correlation coefficient (*P*<0.0001).

### Discriminatory value of the different uPAR variants

#### Domain I; uPAR(I)

uPAR(I) was treated as a binary variable with the limit of quantification (LOQ=20.19 fmol ml^−1^) as the cut-point. The resulting discrimination is shown in Table 2. Performing a general evaluation of the differences between the different groups (type 3 hypothesis) a significant difference was found (*P*=0.008). A repeated measures analysis shows that in the cancer group there are significantly more cases with uPAR(I) levels above the quantification limit than in the group with ONM findings (*P*=0.009), whereas all other comparisons are non-significant. Dichotomizing the data at the 3rd quartile (31.23 fmol ml^−1^) did not result in an improved discrimination (Table 2). Odds ratios were calculated to evaluate the probability of uPAR(I) being elevated in one diagnostic group compared with another. Significant ORs were found when comparing the ONM finding group to the CRC group (Table 3).

#### suPAR (I–III)

Intact suPAR (suPAR(I–III)) was treated as a continuous variable on the log scale, and the discriminatory value was evaluated comparing mean levels for the different diagnostic groups given as the relative difference (Table 4). The type 3-hypothesis test was significant (*P*=0.002). When comparing the CRC group to ONM and ‘no findings’ group's significant relative differences in mean suPAR(I-III) levels were found (20.9% (95% CI:10.2–32.6), *P*<0.0001 and 17.0% (95% CI:5.5–29.7), *P*=0.003, respectively). Comparison of the adenoma and ONM finding groups showed a relative difference of 9.9% ((95% CI: 0.1–21.0), *P*=0.052) All other comparisons were insignificant. Pooling the non-CRC groups and testing for a difference between CRC and this group showed a significant difference (*P*=0.002).

#### suPAR(I–III)+(II–III)

Intact and cleaved suPAR (suPAR(I–III)+(II–III)) was treated as a continuous variable on the log scale, and the discriminatory value was evaluated comparing mean levels for the different diagnostic groups (Table 4). The type 3 hypothesis was significant (*P*=0.004). For suPAR(I–III)+(II–III) a significant difference was found comparing suPAR(I–III)+(II–III) levels in the CRC group to the levels in the ONM finding group (18.5% (95% CI:9.0–28.8), *P*<0.0001). The relative difference between the CRC patients and subjects with no findings was 11.3% (95% CI: 0.1–24.0, *P*=0.052), for subjects with adenomas compared to those with ONM finding the difference was 8.9% (95% CI: 0.8–19.5, *P*=0.072). All other comparisons were insignificant. Pooling the groups presenting no CRC and testing for a difference between the CRC group and this group showed a significant difference (*P*=0.007).

#### Proportional analyses of all uPAR variants

The proportion of patients presenting higher uPAR values than patients in the other diagnosis groups was analysed for each case, and for the M0 (=no distant metastasis diagnosed) CRC patients, respectively. Results are given in Table 5.

## Discussion

In this case–control study, plasma levels of the different uPAR variants were shown to increase with severity of each diagnostic group. For all three markers significant higher levels were seen in the CRC group compared with the uPAR levels in the other three diagnostic groups, suggesting that the markers used in this study may be potential discriminators in the early detection of colorectal cancer. Even though none of the markers were cancer specific the suPAR variants should not be excluded as potential tools in the detection and classification of CRC patients. Previous studies have shown diagnostic value of uPAR variants when combined with other serological markers in both ovarian and prostate cancer (Piironen *et al*, 2006; Henic *et al*, 2008).

In this study the number of patients with detectable levels of uPAR(I) was shown to be significantly lower in the diagnostic group ‘ONM findings’ compared with the CRC and adenoma groups. For the levels of suPAR(I–III)+(II–III) significant differences were only seen when comparing the groups CRC and ‘ONM findings’. Therefore uPAR(I), when compared with other soluble uPAR forms, may be superior as a diagnostic marker in CRC. When this was analysed by a proportional analysis of each case compared with matched patients from the other diagnosis groups (Table 5), uPAR(I) were not superior to the two other uPAR variants studied. However, there are strong indications that the blood level of uPAR(I) reflects the activity of uPA (Zhou *et al*, 2000). Thus high levels are indicative of high uPA activity, leading to plasmin formation and breakdown of the extracellular matrix, which is a prerequisite for cancer invasion (Dano *et al*, 2005). Notably levels of uPAR(I) have previously been shown to be of interest both in cancer detection and as a prognostic marker (Almasi *et al*, 2005; Piironen *et al*, 2006; Henic *et al*, 2008).

In all analyses of the different uPAR forms, the CRC group presented significantly higher values than the remaining groups. This observation supports the hypothesis that suPAR levels in blood are increasing during carcinogenesis (Piironen *et al*, 2006; Henic *et al*, 2008) and despite the insignificant differences between the adenoma group and ‘ONM/no findings’ groups the hypothesis was supported by a ‘trend’ going towards the level of significance when comparing these groups (see Table 4). As shown in Table 1 all diagnoses in the adenoma group could not be certified pathologically, a fact that may bias our findings. In addition, it is well established that some adenomas have higher risk of transforming into carcinomas than others (Bond, 2003). Owing to data limitations differences in uPAR levels were not studied according to adenoma type or size, variables that are indeed of great interest when studying potential screening markers in CRC.

For all markers the most significant differences between groups were found when comparing the diagnostic groups: CRC and ‘ONM finding’. Initially the group of ‘no finding’, characterized by having no reported co-morbidity and no findings at endoscopical examination, were expected to present with the lowest levels of the different uPAR forms and hence the most significant differences compared with CRC. When interpreting the results of this study, it should be noted that all included subjects were symptomatic and when looking at the actual mean values for the two groups: no findings and ‘ONM’ findings they were only slightly different −3.3% for suPAR (I–III) and −6.5% for suPAR (I–III)+(II–III). The diagnostic group of ‘ONM finding’ appeared to be very homogeneous and – with one exception – only included subjects presenting benign lesions of the bowel wall (diverticulosis and haemorrhoids). suPAR has been reported to be elevated in different cancers, but whether suPAR is elevated in other systemic diseases is not well described. Total suPAR has been shown to be elevated in patients with infectious and inflammatory diseases; hence suPAR levels may be elevated because of co-morbidity. Patients could have systemic disease at the time of the examination that were not registered and hence present increased levels of uPAR.

All soluble uPAR forms were shown to be markers of interest in the detection of CRC despite the fact that they were not cancer specific. These findings lead to the hypothesis that including these markers in a multi-marker profile could be beneficial in the detection and classification of CRC when combined with other markers and clinical variables, as previously shown in prostate and ovarian cancer.

A sufficiently powered study including other variables that may influence the uPAR levels (co-morbidity, life style variables) could be conducted measuring uPAR levels in the original study population of more than 5000 individuals. This would allow for an evaluation of the possibility of identifying individuals with a high risk for CRC adjusted for relevant clinical variables and to evaluate uPAR levels in association with other serological biomarkers such as carcinoembryonic antigen and plasma tissue inhibitor of metalloproteinases-1 (Nielsen *et al*, 2007).

## Figures and Tables

**Table 1:** Subject characteristics for each case/controls with 77 individuals in each group

	**Subjects, *n* (%)**
*Gender*	
Female	37 (48)
Male	40 (52)
	
*Age group*	
40+	3 (4)
50+	10 (13)
60+	17 (22)
70+	26 (34)
80+	21 (27)
	
*Localisation*	
Right colon	23 (30)
Left colon	34 (44)
Rectum	20 (26)
	
*Cancer stage*	
I	9 (12)
II	32 (42)
III	16 (21)
IV	15 (19)
not specified	5 (6)
	
*Adenomas*	
Tubular	45 (58)
Tubulovillous	16 (21)
Villous	1 (1)
Serrat	1 (1)
Not specified	14 (18)
	
*Size*	
<1 cm	41 (53)
>1 cm	33 (43)
Not specified	3 (4)
	
*Other non-malignant finding*	
Diverticulosis	72 (94)
Haemorrhoids	4 (5)
Inflammatory bowel disease	1 (1)

**Table 2:** uPAR(I) treated as a binary variable with cut-point at the limit of quantification or the 3rd quartile

**Diagnostic group**	**Limit of quantification + uPAR(I), *n* (%)**	**>3rd quartile + uPAR(I), *n* (%)**
CRC	46 (59.7)	25 (32.5)
Adenoma	36 (46.8)	18 (23.4)
Other non-malignant finding	28 (36.4)	12 (15.6)
No finding	38 (49.4)	22 (28.6)

The table shows the proportion of subjects with detectable uPAR(I) in each diagnosis group (*n*=77).

**Table 3:** The odds ratios (OR) for elevated uPAR(I) comparing diagnostic groups.(ONM=other non-malignant)

**Groups**	**Odds ratio and (95% confidence limits)**	***P*-value**
ONM finding *vs* no finding	0.50 (0.25–0.99)	0.047
Adenoma *vs* no finding	1.18 (0.62–2.23)	0.611
Adenoma *vs* ONM finding	2.38 (1.27–4.47)	0.007
CRC *vs* adenoma	1.45 (0.79–2.63)	0.235
CRC *vs* no finding	1.71 (0.91–3.18)	0.093
CRC *vs* ONM finding	3.44 (1.86–6.37)	<0.0001

**Table 4:** Relative mean difference (%) between the diagnostic groups for intact suPAR and intact+cleaved suPAR

**Marker**	**Groups**	**Relative mean difference and (95% confidence limits in %)**	***P*-value**
suPAR(I–III)	Non-malignant *vs* No	−3.3 (−13.3–5.8)	0.488
*P*=0.002^a^	Adenoma *vs* no finding	6.4 (−3.4–17.2)	0.208
	Adenoma *vs* ONM finding	9.9 (−0.1–21.0)	0.052
	CRC *vs* adenoma	9.0 (−1.5–18.5)	0.089
	CRC *vs* no finding	17.0 (5.5–29.7)	0.003
	CRC *vs* ONM finding	20.9 (10.2–32.6)	<0.0001
suPAR(I–III)+(II–III)	Non-malignant *vs* No	−6.5 (−17.0–3.1)	0.190
*P*=0.004^a^	Adenoma *vs* no finding	2.3 (−7.2–12.7)	0.653
	Adenoma *vs* ONM finding	8.9 (−0.8–19.5)	0.072
	CRC *vs* adenoma	8.1 (−1.9–17.2)	0.110
	CRC *vs* no finding	11.3 (−0.1–24.0)	0.052
	CRC *vs* ONM finding	18.5 (9.0–28.8)	<0.0001

a Results of type III hypothesis tests (ONM=other non-malignant).

**Table 5:** Showing the proportion of cancer patients with uPAR levels exceeding the level found in the corresponding adenoma, ONM and no finding was estimated with 95% CI

	**Subset**	**Adenoma (95% CI)**	**Other (95% CI)**	**None (95% CI)**
suPAR( I–III)+(II–III)	All	64 (53–74)	70 (60–80)	66 (56–77)
	M0	59 (44–73)	67 (54–80)	61 (48–75)
suPAR(I–III)	All	55 (43–66)	70 (60–80)	64 (53–74)
	M0	51 (37–65)	67 (54–80)	61 (48–75)
uPAR(I)^a^	All	61 (49–73)	67 (55–80)	60 (48–72)
	M0	54 (38–70)	68 (49–83)	59 (44–75)

M0=analyses restricted to the group of patients presenting no distant metastases (*n*=49).

a Ties excluded.
